# MicroRNA-32 promotes cell proliferation, migration and suppresses apoptosis in breast cancer cells by targeting FBXW7

**DOI:** 10.1186/s12935-017-0383-0

**Published:** 2017-01-25

**Authors:** Wei Xia, JueYu Zhou, HaiBo Luo, YunZhou Liu, CanCan Peng, WenLing Zheng, WenLi Ma

**Affiliations:** 1 Institute of Genetic Engineering, Southern Medical University, Guangzhou, 510515 People’s Republic of China; 2 Department of Clinical Laboratory, No.421 Hospital of PLA, Guangzhou, People’s Republic of China

**Keywords:** MiR-32, Breast cancer, FBXW7, Proliferation, Apoptosis

## Abstract

**Background:**

MicroRNAs are a class of small non-coding RNAs that are involved in many important physiological and pathological processes by regulating gene expression negatively. The purpose of this study was to investigate the effect of miR-32 on cell proliferation, migration and apoptosis and to determine the functional connection between miR-32 and FBXW7 in breast cancer.

**Methods:**

In this study, quantitative RT-PCR was used to evaluate the expression levels of miR-32 in 27 breast cancer tissues, adjacent normal breast tissues and human breast cancer cell lines. The biological functions of miR-32 in MCF-7 breast cancer cells were determined by cell proliferation, apoptosis assays and wound-healing assays. In addition, the regulation of FBXW7 by miR-32 was assessed by qRT-PCR, Western blot and luciferase reporter assays.

**Results:**

MiR-32 was frequently overexpressed in breast cancer tissue samples and cell lines as was demonstrated by qRT-PCR. Moreover, the up-regulation of miR-32 suppressed apoptosis and promoted proliferation and migration, whereas down-regulation of miR-32 showed an opposite effect. Dual-luciferase reporter assays showed that miR-32 binds to the 3′-untranslated region of FBXW7, suggesting that FBXW7 is a direct target of miR-32. Western blot analysis showed that over-expression of miR-32 reduced FBXW7 protein level. Furthermore, an inverse correlation was found between the expressions of miR-32 and FBXW7 mRNA levels in breast cancer tissues. Knockdown of FBXW7 promoted proliferation and motility and suppressed apoptosis in MCF-7 cells.

**Conclusions:**

Taken together, the present study suggests that miR-32 promotes proliferation and motility and suppresses apoptosis of breast cancer cells through targeting FBXW7.

## Background

Breast cancer is one of the most common malignant diseases in women around the world and can be seriously harmful to women’s health and survival [1–3]. In more than 90% of patients with breast cancer initial invasion and distant metastasis are the major causes of death. The pathogenesis of breast cancer remains largely unknown. Molecular biology studies on breast cancer have shown that it is a complex process with multi-gene and multi-factor interactions that are mutually influenced like other malignancies [4, 5].

MicroRNAs (miRNAs) are a class of evolutionarily conserved, endogenous, small, about 20–22 nt nucleotides in length, non-coding single-stranded RNAs that regulate gene expression by targeting the 3′ untranslated region (3′-UTR) of mRNAs [6, 7]. A growing number of studies have indicated that miRNA expression is associated with various tumors, including breast cancer [8–10]. MiR-32 is located on the 14th intron of gene C9orf5 [11]. Recently, miR-32 has been identified as an important regulator in tumorigenesis and it may act as a tumor suppressor or an oncogene in different cancers [12–14]. Previous studies have shown that miR-32 was dysregulated in breast cancer although its biological roles remain unclear [15, 16].

FBXW7 is one of the F-box protein family members, which can specifically identify and degrade substrate proteins in the ubiquitin–proteasome system (UPS). It is important in cell cycle regulation, transcriptional regulation, apoptosis and cell signal transduction, and is characterized by an approximately 40-amino acid motif, the F-box. Recent studies have shown that FBXW7 is believed to be a tumor suppressor that targets various oncogenic proteins [17, 18]. FBXW7 mutations and deletions can cause chromosome instability and accelerate the accumulation of cancer cell proliferation associated genes such as Myc, CyclinE and Aurora-A [19, 20]. FBXW7 has been identified in diverse human cancers, including T cell acute lymphoblastic leukemia, pancreatic cancer, endometrial cancer, and colon cancer [21–23]. The study of FBXW7 is very important to understand the mechanism of tumorigenesis and to provide new targets for cancer diagnosis and treatment.

Based on the information above, we demonstrated that miR-32 was highly expressed in both breast cancer tissues and cell lines, and promoted the proliferation and migration and suppressed apoptosis of breast cancer cells. Moreover, we verified that miR-32 directly targeted FBXW7 through binding to the FBXW7-3′-UTR. Depletion of FBXW7 by shFBXW7 could promote the proliferation and motility,supress apoptosis of breast cancer cells. Furthermore, our results imply that high expression of miR-32 may contribute to the development of breast cancer through targeting FBXW7. Our findings will help to elucidate the functions of miR-32 and its role in breast cancer tumorigenesis.

## Methods

### Tissue samples

Breast cancer tissues and adjacent normal breast tissues were obtained from 27 patients at the Nanfang Hospital of the Southern Medical University from February 2013 to March 2015. All samples were snap-frozen in liquid nitrogen immediately after surgical removal and no patient had received chemotherapy or radiotherapy before surgery. The study was approved by the Ethics Committee of the Nanfang Hospital of the Southern Medical University and samples were obtained with informed consent from all patients. Clinical pathological characteristics of all patients are summarized in Table 1.

**Table 1:** Association of FBXW7 mRNA or miR-32 expression with clinicopathological data from breast cancer patients by quantitative PCR

Characteristics	N	miR-32		FBXW7	
		Relative expression level	P value	Relative expression level	P value
Age (year)					
≤50	16	3.84 ± 2.84	0.100	1.11 ± 1.29	0.147
>50	11	2.26 ± 1.37		0.49 ± 0.42	
Tumor size (cm)					
≤2	5	3.05 ± 0.59	0.969	0.56 ± 0.46	0.631
>2 and ≤5	14	3.31 ± 3.13		0.66 ± 0.71	
>5	8	3.08 ± 1.94		1.01 ± 1.34	
ER status					
Negative	7	4.05 ± 4.26	0.509	1.08 ± 1.48	0.454
Positive	20	2.89 ± 1.45		0.62 ± 0.63	
PR status					
Negative	7	1.88 ± 0.90	0.100	1.18 ± 1.42	0.324
Positive	20	3.65 ± 2.66		0.59 ± 0.64	
HER-2 status					
Negative	3	2.10 ± 1.41	0.885	0.38 ± 0.09	0.479
Positive	24	3.22 ± 2.57		0.79 ± 0.96	
Lymph node status					
Negative	12	3.82 ± 3.36	0.24	0.70 ± 1.14	0.817
Positive	15	2.69 ± 1.28		0.78 ± 0.73	
Clinical stage					
I–II	19	3.32 ± 2.85	0.695	0.85 ± 1.05	0.369
III–IV	8	2.90 ± 1.13		0.49 ± 0.44	
Ki-67					
Negative	2	3.27 ± 1.23	0.965	0.54 ± 0.23	0.749
Positive	25	3.19 ± 2.53		0.76 ± 0.95	

### Cell culture, construct and transfection

The normal human breast cell line MCF-10A and breast cancer cell lines MCF-7 and MDA-MB-231 were obtained from the Chinese Academy of Sciences Cell Bank (Shanghai, China). MCF-10A was incubated in DMEM/F12 (1:1) (Hyclone, USA) with 10% fetal bovine serum (FBS, Hyclone). All breast cancer cells were cultured in DMEM (Hyclone, USA) supplemented with 10% FBS. Cells were cultured at 37 °C in a humidified atmosphere with 5% CO_2_. The miR-32 mimic, inhibitor, mimic-negative control (NC), inhibitor-NC and shFBXW7 oligo were obtained from GenePharma (Shanghai, China) and transfected into MCF-7 cells using Lipofectamine 2000 (Invitrogen, Carlsbad, CA, USA) according to the manufacturer’s instructions. The RNA concentration of each transfection was 50 nM.

### RNA isolation and real-time PCR detection

Total RNA was extracted from tissues and cells using TRIzol reagent (Invitrogen, California, USA) and reversely transcribed into cDNA according to the manufacturer’s instruction. Real-time PCR detection of miR-32 was conducted as reported by Francesca Fornari et al. [24]. Detection of FBXW7 was performed via PrimeScript^TM^RT reagent Kits (TaKaRa), and Permix Ex TaqII (TaKaRa) according to the manufacturer’s protocol. The primers used for real-time PCR detection include: miR-32-forward, 5′-GCG GCG TAT TGC ACA TTA CT-3′, and reverse, 5′-TCG TAT CCA GTG CAG GGT C-3′, U6-forward, 5′-CTC GCT TCG GCA GCA CA-3′, and reverse, 5′-AAC GCT TCA CGA ATT TGC GT-3′; FBXW7-forward, 5′-CCA CTG GGC TTG TAC CAT GTT-3′ and reverse, 5′-CAG ATG TAA TTC GGC GTC GTT-3′; β-actin-forward, 5′-TGA CGT GGA CAT CCG CAA AG-3′, and reverse, 5′- CTG GAA GGT GGA CAG CGA GG-3′. MiR-32 level was normalized with U6 and FBXW7 level was normalized with β-actin.

### MTT assay

Cell proliferation was observed by the MTT assay. MCF-7 cells (1 × 10^4^) were plated into 96-well plates. Cell proliferation was assessed at the indicated time points after transfection as follows: 50 μl MTT (Keygen) working solution was added into the wells and the cells were incubated for 4 h at 37 °C, followed by removal of the culture medium and addition of 150 μl DMSO for 20 min. The absorbance at 550 nm was measured in a Tecan Infinite Multiskan (Swiss).

### Cell apoptosis assay

MCF-7 cells (1 × 10^5^) were plated into 6-well plates and transfected with miR-32 mimic/inhibitor or mimic-NC/inhibitor-NC. Forty-eight hours after transfection, cells were harvested and resuspended in 500 μl binding buffer. The cell suspension was incubated with 5 μl Annexin V-FITC for 15 min in a dark place. 50 μg/ml PI was added to each sample after which flow cytometry (C6; BD Biosciences, Franklin Lakes, NJ, USA) was used to determine apoptosis of the MCF-7 cells.

### Wound-healing assays

The ability of migration of breast cancer cells was investigated by wound-healing assays. After 48 h of miR-32 mimic, inhibitor or mimic-NC/inhibitor-NC transfection, MCF-7 cells were obtained, plated at 8 × 10^4^ cells/well in 24 well plates and cultured until they formed a confluent monolayer. Wounds were scratched by 10 μl pipette tips. The MCF-7 cells were washed 3 times with PBS and then cultured in serum-free medium and photographed every 12 h by using QImagine Software.

### Luciferase reporter assay

The 3′-UTR of FBXW7 mRNA which contains a putative target region for miR-32, was amplified from genomic DNA by PCR. The FBXW7 3′-UTR mutant construct was generated by overlap extension PCR. Fragments were inserted between the *Xho*I and *Not*I sites in the psiCHECK™-2 Dual Luciferase miRNA target expression vector (Promega, USA) to generate the recombinant vectors FBXW7-wt and FBXW7-mut. Both insertions were verified by sequencing (Sangon, China). Co-transfection of the reporter vectors and miRNA (miR-32 mimic or mimic NC) was performed in MCF-7 cells using Lipofectamine 2000. Forty-eight hours after transfection, luciferase and renilla signals were measured using the Dual Luciferase Reporter Assay Kit (Promega) according to the manufacturer’s protocol.

### Western blotting

Cells were washed twice with Hanks’ balanced salt solution and lysed in RIPA lysis buffer (50 mM Tirs-Cl, pH 7.4, 120 mM NaCl, 1% NP-40, 0.2% SDS, 1 mM EDTA and complete protease inhibitor), and centrifuged for 20 min at 13,000*g*, 4 °C. The protein concentrations were subsequently determined using a BCA Protein Assay Kit (Beyotime, China) according to the manufacturer’s instructions. Protein samples (20 µg) were denatured with 4× loading buffer (TAKARA) at 95 °C for 5 min. The polyvinylidene difluoride (PVDF; Life Technologies) membrane was blocked with phosphate-buffered saline (PBS) supplemented with 5% non-fat milk. Equal quantities of protein were subjected to SDS-PAGE and gels were transferred onto PVDF membranes. The PVDF membrane was then incubated with the following anti-bodies: Anti-FBXW7 (1:500; Abcam) and anti-β-actin (1:2000; Cell Signaling Technology), at 4 °C overnight. The PVDF membrane was washed three times with PBS containing 5% Tween (Sigma-Aldrich), and incubated with horseradish peroxidase-conjugated rabbit anti-mouse secondary antibody at room temperature for 2 h. An ECL kit was used to visualize the protein bands according to the manufacturer’s instructions. The relative protein expression levels were analyzed using Image-ProPlus 6.0 software (Media Cybernetics, Inc., Rockville, MD, USA).

### Statistical analysis

All statistical analyses were carried out using the SPSS 18.0 statistical software package. Continuous variables were expressed as mean ± SD. Differences between groups were calculated with the Student’s t test. Pearson’s correlation was used to estimate the relationship between expression levels of miR-32 and FBXW7 mRNA. A two-tailed P value test accompanied by a P value <0.05 was considered statistically significant.

## Results

### Expression of miR-32 is greatly increased in breast cancer tissues and cell lines

**Fig. 1:**
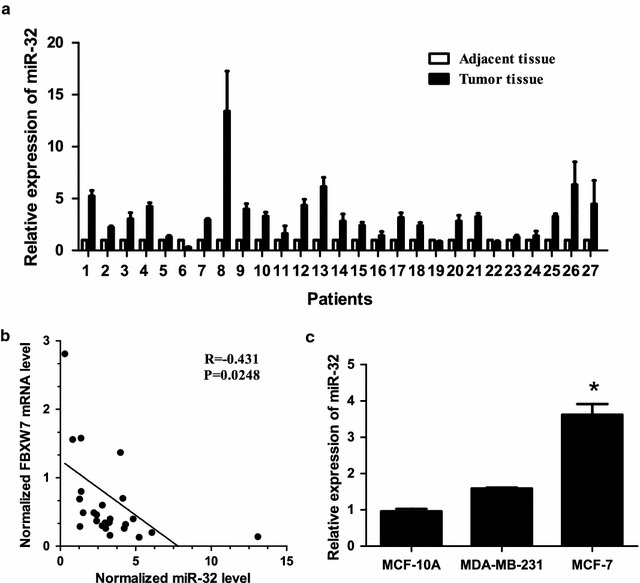
MiR-32 is up-regulated in breast cancer samples and cell lines. **a** Expression levels of miR-32 were up-regulated in 20 breast cancer tissues when compared with adjacent normal tissues. **b** In 19 breast cancer tissues and corresponding adjacent non-tumor tissues, miR-32 has a negative correlation with FBXW7 mRNA expression. **c** The expression level of miR-32 was up-regulated in MCF-7 and MDA-MB-231 cell lines when compared with MCF-10A. *Bars* represent the mean of three independent experiments performed three times ±SD. *P < 0.01

In order to evaluate the potential involvement of miR-32 in breast cancer, the expression level of miR-32 was detected in 27 breast cancer tissues and corresponding adjacent non-tumor tissues by real-time PCR. As shown in Fig. 1a, miR-32 expression was greatly increased in 20/27 breast cancer tissues when compared with adjacent non-tumor tissues (74.07%, P < 0.05). The correlation between miR-32 expression levels and clinical pathological characteristics are summarised in Table 1. In all specimens tested, we found an inverse correlation between the expression of miR-32 and the level of FBXW7 mRNA (Fig. 1b, r = −0.431, P = 0.0248). We also found that miR-32 was significantly elevated in tested breast cancer cell lines when compared with normal breast cell line MCF-10A (Fig. 1c). These results indicated that the expression of miR-32 was up-regulated in breast cancer.

### MiR-32 promotes proliferation and migration of breast cancer cells

**Fig. 2:**
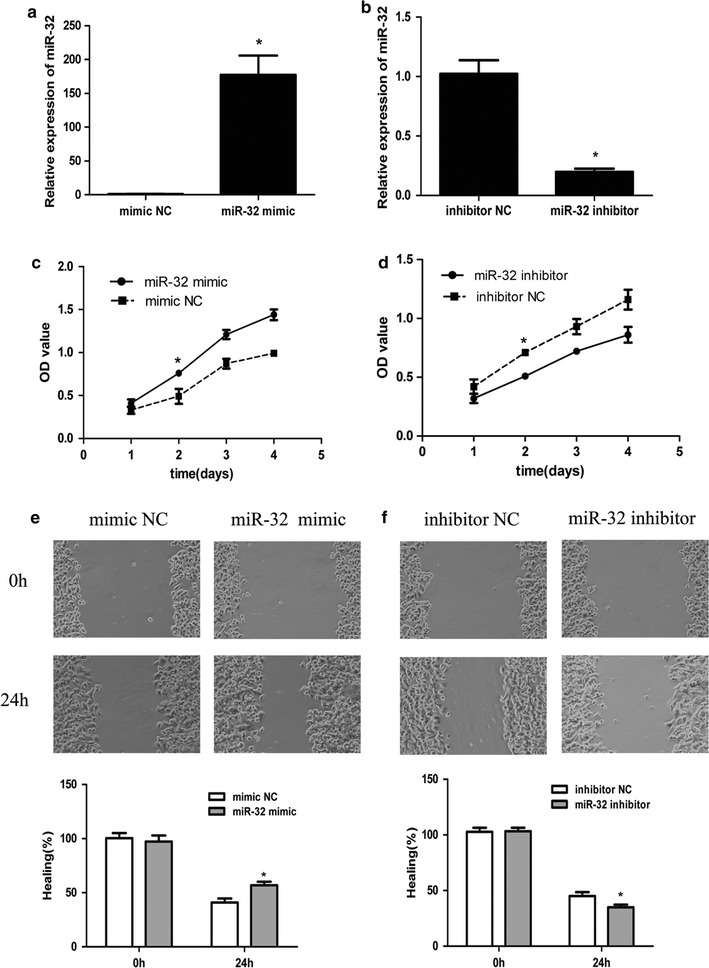
Effect of miR-32 expression on growth and migration in breast cancer cells. **a**, **b** QRT-PCR of miR-32 expression in mimic/inhibitor or NC transfected MCF-7 cells. U6 was used as an internal control. **c**, **d** MTT analysis of MCF-7 growth following transfection with miR-32 mimic/inhibitor or NC. MiR-32 mimic promotes cell proliferation and inhibitor suppresses cell growth. **e**, **f** Wound-healing assay showing that gain of miR-32 promotes cell migration and loss of miR-32 suppresses cell migration. Each assay was repeated three times. *P < 0.05

In order to explore the involvement of miR-32 in breast cancer development and progression, miR-32 mimic, inhibitor or NC oligonucleotides were transfected into MCF-7 cells to restore its expression level as tested by real-time PCR (Fig. 2a, b). The impact of miR-32 on cell proliferation and viability was analyzed by MTT assay. As shown in Fig. 2c, cells transfected with miR-32 mimic showed significantly higher optical density (OD) values at 550 nm than the NC group from day 2 until day 4, in a time dependent manner. The miR-32 inhibitor group displayed lower OD values and cell proliferation was significantly suppressed in this group (Fig. 2d). The effect of miR-32 on the migration of breast cancer cells was characterized by a wound-healing assay, which showed that up-regulation of miR-32 could significantly promote cell migration and down-regulation of miR-32 could suppress cell migration (Fig. 2e, f). Taken together, miR-32 is able to promote the proliferation and migration of breast cancer cells in vitro.

### MiR-32 inhibits apoptosis of breast cancer cells

**Fig. 3:**
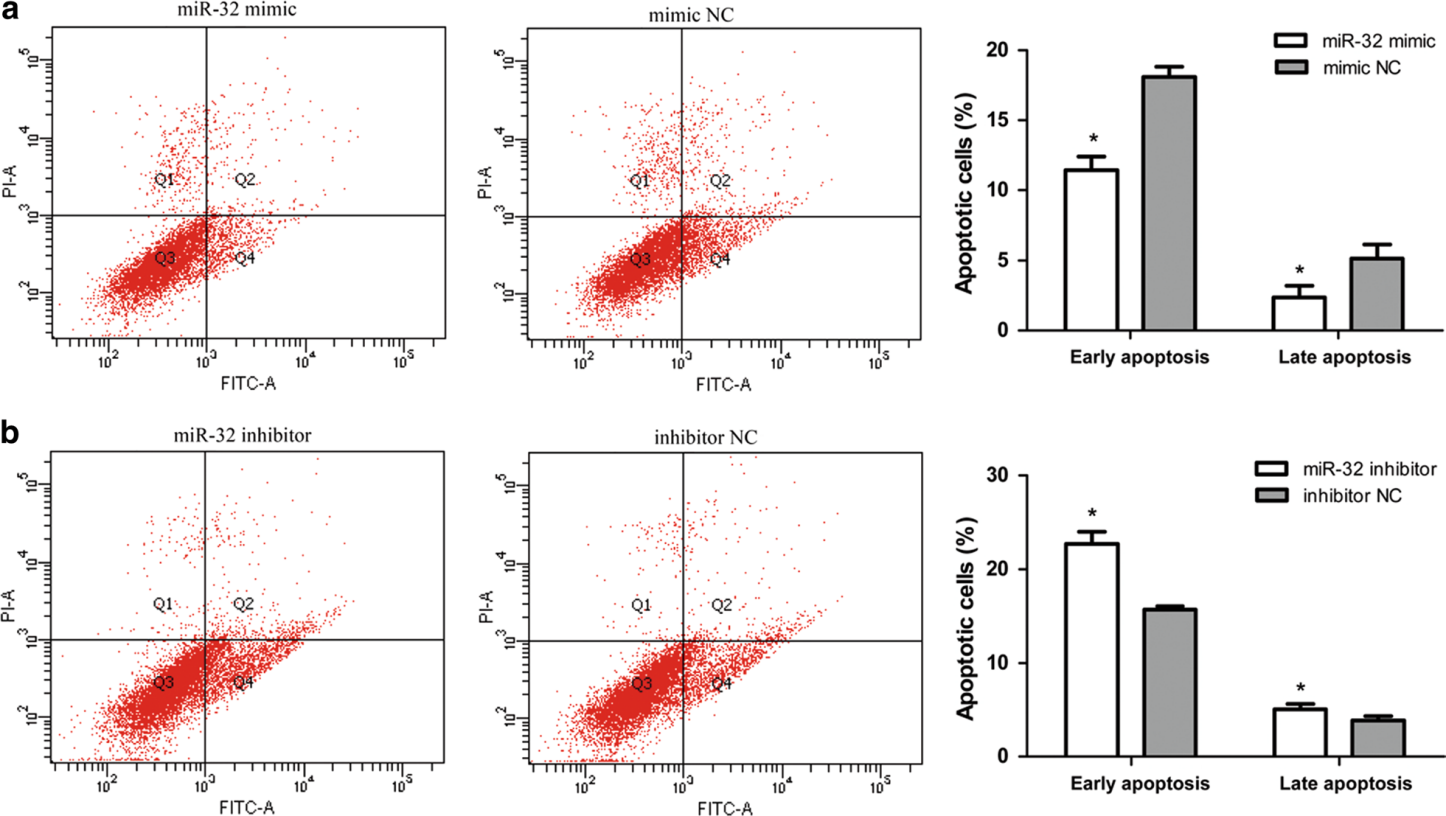
Effects of miR-32 expression on apoptosis in breast cancer cells. **a**, **b** MiR-32 mimic suppresses MCF-7 cell apoptosis and miR-32 inhibitor induces cell apoptosis. Cell apoptosis was analyzed by Annexin V-FITC analysis. Data represents the mean of three independent assays ±SD. *P < 0.05

MiR-32 mimic, inhibitor or NC miRNA oligonucleotides were transfected into MCF-7 cells and apoptosis was determined by FACS analysis. As shown in Fig. 3a, apoptosis in miR-32 mimic transfected cells was significantly inhibited when compared with the NC group. Down-regulation of miR-32 had the opposite effect (Fig. 3b). These results suggested that miR-32 may affect apoptotic pathways in regulating tumorigenicity.

### FBXW7 is a novel target gene of miR-32 in MCF-7 cells

**Fig. 4:**
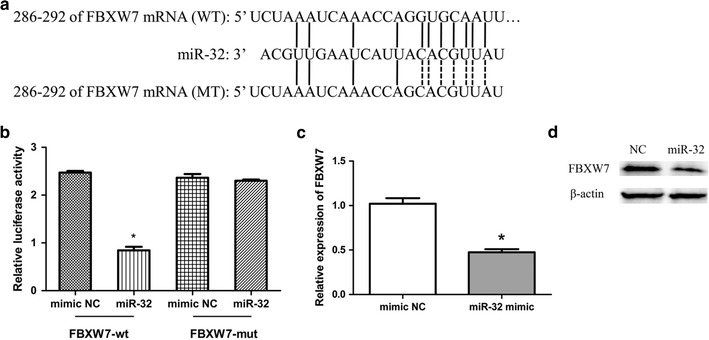
MiR-32 targeted FBXW7. **a** The miR-32 binding site in FBXW7 3′-UTR, located 286-292 bp upstream of the FBXW7 3′-UTR. **b** The relative luciferase activity in miR-32 mimic group and NC group. **c** The relative expression of FBXW7 in miR-32 mimic transfected group and NC group. Bars represent the mean of three independent experiments performed three times ±SD. **d** Western blot analysis of FBXW7 protein levels in miR-32 overexpressing and NC cells. *P < 0.05

To find the underlying mechanisms of miR-32 in breast cancer, we investigated potential targets of miR-32 using prediction software (TargetScan, PicTa, miRanda, the miRBase, DIANA TarBase). Bioinformatic analyses predicted that FBXW7 was a potential target of miR-32. To validate whether miR-32 might mediate the decay of FBXW7 mRNA via the 3′-UTR, we subcloned FBXW7 mRNA 3′-UTR into psiCHECK™-2 luciferase reporter construct (FBXW7-wt). A mutant form of FBXW7 mRNA 3′-UTR was generated through mutating the binding site of miR-32 (FBXW7-mut) (Fig. 4a). Next, these constructs were transfected into MCF-7 cells with miR-32 mimic or NC miRNA. The luciferase assay showed that miR-32 remarkably down-regulated the luciferase activity of the FBXW7-wt construct, while the luciferase activity of FBXW7-mut in MCF-7 cells was unchanged (Fig. 4b). As shown in Fig. 4c, d, FBXW7 expression was significantly decreased in miR-32 mimic transfected cells when compared with the NC group whatever the mRNA or protein level. These data demonstrated that miR-32 can down regulate FBXW7 expression by directly targeting its 3′-UTR.

### FBXW7 inhibits cell growth and cell migration

**Fig. 5:**
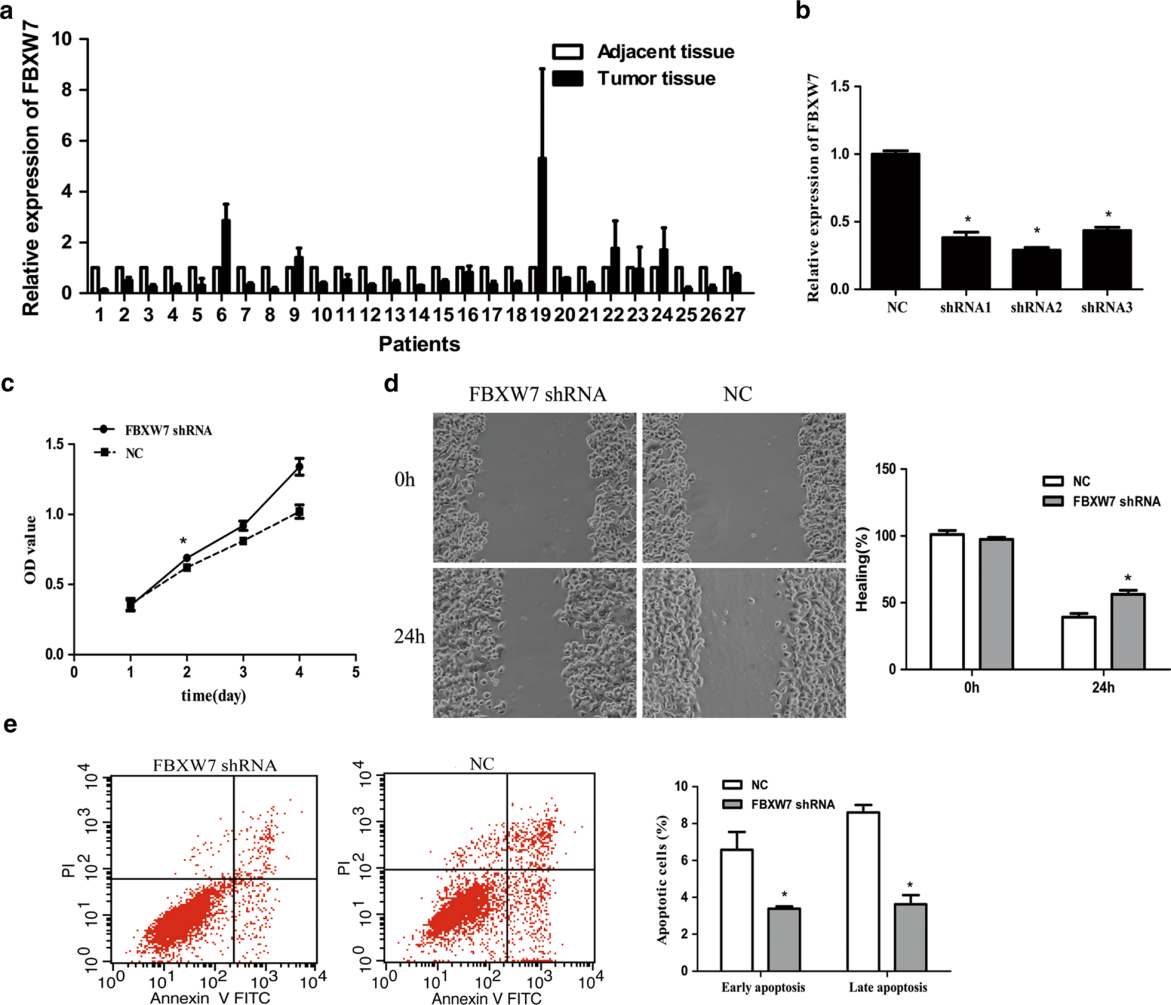
The effect of FBXW7 depletion in MCF-7 cells. **a** Expression level of FBXW7 was down-regulated in 19 breast cancer tissues compared with adjacent normal tissues. **b** The expression level of FBXW7 in transfected shRNA MCF-7 cells. **c** MTT analysis showed that FBXW7 depletion promotes cells proliferation. **d** Wound-healing assay showed that loss of FBXW7 promotes cell migration. **e** FBXW7 depletion suppresses MCF-7 cell apoptosis. Data represents the mean of three independent assays ±SD. *P < 0.05

We also evaluated the expression of FBXW7 in 27 paired breast cancer and adjacent normal breast tissues by qRT-PCR. Data showed that the average level of FBXW7 mRNA was significantly decreased in 19/27 breast cancer tissues when compared with corresponding normal tissues (70.37%, P < 0.05) (Fig. 5a). Moreover, FBXW7 mRNA levels in breast cancer cases were inversely correlated with miR-32 expression. MCF-7 cells were transfected with FBXW7 shRNA or NC to further investigate whether the effect of miR-32 on cell proliferation, migration and apoptosis of MCF-7 cells was via targeting FBXW7 (Fig. 5b). As shown in Fig. 5c, the MTT value of cells transfected with shRNA was significantly higher than that of cells transfected with NC 2 days post-transfection. The results showed that suppression of FBXW7 expression by shRNA promoted cell migration and inhibited cell apoptosis when compared with the NC group (Fig. 5d, e). Altogether, our findings suggested that miR-32 promotes cell growth and migration of breast cancer cells, at least in part, by targeting FBXW7.

### Interference of FBXW7 restored miR-32-mediated breast cancer cell growth and migration

**Fig. 6:**
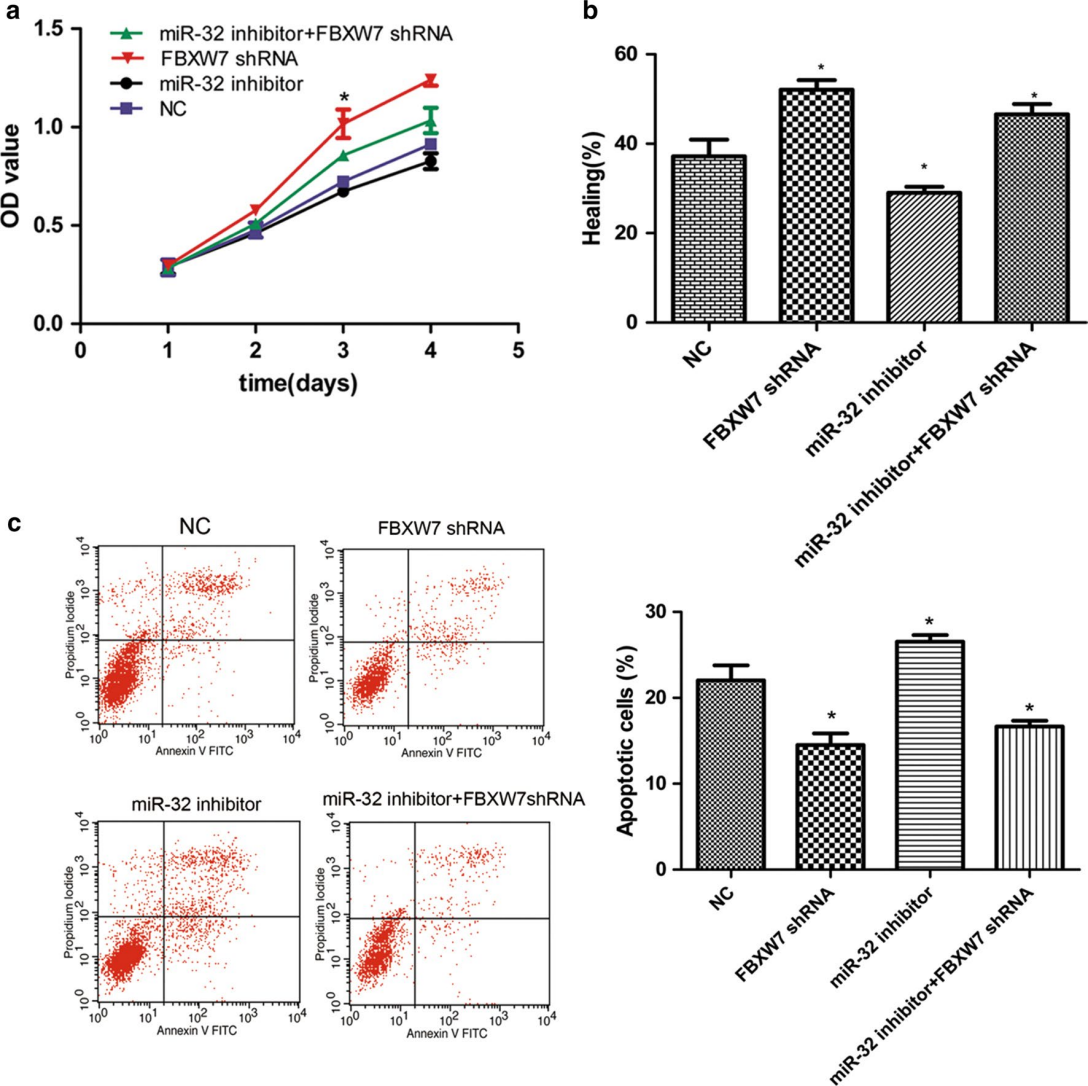
The effect of FBXW7 depletion on miR-32-mediated breast cancer chemoresistance. **a** The proliferation rate of MCF-7 cells were detected through MTT assay at different time periods after transfection of miR-32 inhibitor and FBXW7 shRNA independently or simultaneously compared with NC group. **b** Statistical analysis of the proportion of healing compared with NC group. **c** Annexin V/PI analysis was performed to determine cell apoptosis in the indicated groups. Data represents the mean of three independent assays ±SD. (*P < 0.05)

To this end, the FBXW7-targeting shRNA oligo were employed to deplete endogenous FBXW7 in breast cancer cells. NC, miR-32 inhibitor and FBXW7 shRNA oligo were transfected independently or simultaneously into MCF-7 cells. As shown in Fig. 6a, b, depletion of FBXW7 by transfection of shFBXW7 oligo significantly promote the proliferation and migration of MCF-7 cells compared with NC group. However, the proliferation and migration of MCF-7 cells were restored after co-transfection with miR-32 inhibitor. Then, we found that MCF-7 cells exhibited distinct responses to FBXW7, with regard to miR-32-mediated anti-apoptotic effect. Whereas depletion of FBXW7 restored the apoptotic response in miR-32-inhibiting MCF-7 cells (Fig. 6c). These findings inferred that miR-32 promotes cell proliferation, migration, and suppresses apoptosis by inhibiting the expression of FBXW7.

## Discussion

Since dysregulation of specific miRNAs have been found in tumor biopsies and body fluids, accumulating studies have revealed that miRNAs can function as tumor suppressors or oncogenes in the development and progression of breast cancer [25–27].

It has been observed that high miR-32 levels were present in some tumor but low levels in others. For example, miR-32 in lung cancer is significantly down regulated [28], on the contrary, in renal and prostate cancer tissue the level of miR-32 is significantly raised and associated with the prognosis of patients [29, 30]. Moreover, it is believed that mature miR-32 has divergent effects on the development of cancer, such as the elevated expression of miR-32 significantly inhibits the proliferation, migration and invasion of the SGC-7901 gastric cancer cell line [12]. Two other studies showed that miR-32 suppresses osteosarcoma cell proliferation and invasion through regulating Sox9 expression [13] and promotes CRC cells proliferation, migration, and invasion and reduces apoptosis by targeting PTEN [14]. In conclusion, miR-32 may play a different role in different tumor types. In this study, we described the biological significance and the effects of miR-32 dysregulation on cell proliferation, migration and apoptosis in human breast cancer cells. Furthermore, we identified FBXW7 as a direct target of miR-32 in breast cancer cell lines.

F-box and WD-40 domain protein 7 (FBXW7) is one of the F-box protein family members, which can identify the target proteins of the SCF ubiquitin ligase [31]. It plays an important role in many physiological and pathological processes, such as cell growth, differentiation, apoptosis, and cellular signal transduction [32]. Recent studies have demonstrated that FBXW7 is a tumor suppressor. Deletions and mutations of FBXW7 have been identified in a variety of cancers including colorectal cancer, gastric cancer, ovarian cancer, neuroglioma and breast cancer [23, 33]. Iwatsuki et al. [34] found that a low expression of FBXW7 correlated with a poor prognosis in colorectal cancer patients and indicated that FBXW7 could serve as a prognostic factor. Milne et al. [35] reported that loss of FBXW7 played a role in gastric carcinogenesis and Li et al. also found that miR-223, which acts as oncogene, regulated FBXW7in human gastric cancer [36]. In addition, Gong et al. [37] demonstrated that miR-25 promoted gastric cancer progression by directly down-regulating FBXW7 expression. FBXW7 can also bind directly to multiple transcriptional activators and proto-oncogenes including cyclin E, c-Myc and Notch for ubiquitination and subsequent degradation [32, 38, 39]. Studies on FBXW7 as tumor suppressor are extremely important to understand tumorigenesis as it can act as a therapeutic target as well as a diagnostic marker in cancers.

In the present study, we have demonstrated that the expression of miR-32 was increased in the majority of breast cancer tissues and in breast cancer cell lines. Further functional analysis suggested the involvement of miR-32 in the progression of breast cancer, and forced expression of miR-32 significantly promoted proliferation and migration as well as repressed cell apoptosis in breast cancer cell line MCF-7. Our results reveal that miR-32 may act as a tumor promoter to participate in the progression of breast cancer. However, we failed to find a correlation between miR-32 and PR, ER and HER2 expression in the tissues of breast cancer patients, the reason might be that miR-32 has nothing to do with the involved molecular pathways of ER and PR. Our data reflect the heterogeneous nature of tumors and indicate that miR-32 functions are tumor-specific.

According to the results of TargetScan and miRanda analysis, we identified FBXW7 as a target gene of miR-32 which was partly verified by the inhibition of FBXW7 mRNA expression in mimic-transfected MCF-7 cells in vitro. We found an inverse correlation between the expression of miR-32 and the level of FBXW7 mRNA in breast cancer tissues. Our study showed that inhibition of FBXW7 mRNA expression could promote the proliferation and migration, and suppress apoptosis of breast cancer cells. The dual luciferase assay further confirmed that FBXW7 was a direct target of miR-32. MiR-32 was sufficiently strong enough to inhibit FBXW7 via direct binding to the FBXW7 3′-UTR region.

There are some limitations of this study that should be noted. The clinical tissue information comes mainly from the clinical stage, cannot cover all molecular subtypes of breast cancer, and it is difficult to reflect the overall heterogeneity and individual differences. As comprehensive data on miR-32 expression is currently unavailable, further studies are needed to reveal the exact role of miR-32 in breast cancer and subtypes in much larger populations. Further functional analyses are also needed to validate the possible utility of miR-32.

## Conclusion

In conclusion, the present study assessed the expression and functions of miR-32 in breast cancer. In addition, miR-32 induced down-regulation of FBXW7 and regulated the proliferation, migration and apoptosis capability of breast cancer cells. These findings indicate that miR-32 may serve as a tumor gene in breast cancer, at least partly via directly targeting FBXW7, and may therefore act as a potential candidate for miRNA-based therapy against breast cancer.

## Abbreviations

FBXW7: F-box and WD-40 domain protein 7
3′-UTR: 3′ untranslated region
miRNAs: microRNAs
